# Protocol for a pharmacogenomic study on individualised antipsychotic drug treatment for patients with schizophrenia

**DOI:** 10.1192/bjo.2021.945

**Published:** 2021-06-29

**Authors:** Yi Su, Hao Yu, Zhiren Wang, Sha Liu, Liansheng Zhao, Yingmei Fu, Yongfeng Yang, Bo Du, Fuquan Zhang, Xiangrong Zhang, Manli Huang, Cailan Hou, Guoping Huang, Zhonghua Su, Mao Peng, Ran Yan, Yuyanan Zhang, Hao Yan, Lifang Wang, Tianlan Lu, Fujun Jia, Keqing Li, Luxian Lv, Hongxing Wang, Shunying Yu, Qiang Wang, Yunlong Tan, Yong Xu, Dai Zhang, Weihua Yue

**Affiliations:** Institute of Mental Health, The Sixth Hospital of Peking University, China; and Key Laboratory of Mental Health, Ministry of Health & National Clinical Research Center for Mental Disorders (Peking University), China; Institute of Mental Health, The Sixth Hospital of Peking University, China; Key Laboratory of Mental Health, Ministry of Health & National Clinical Research Center for Mental Disorders (Peking University), China; and Department of Psychiatry, Jining Medical University, China; Psychiatry Research Center, Beijing HuiLongGuan Hospital, Peking University, China; Department of Psychiatry, First Hospital/First Clinical Medical College of Shanxi Medical University, China; Mental Health Center, West China Hospital, Sichuan University, China; Shanghai Mental Health Center, Shanghai Jiaotong University, China; Henan Mental Hospital, The Second Affiliated Hospital of Xinxiang Medical University, China; Hebei Mental Health Center, The Sixth People's Hospital of Hebei Province, China; Wuxi Mental Health Center, Nanjing Medical University, China; Department of Geriatric Psychiatry, Nanjing Brain Hospital Affiliated to Nanjing Medical University, China; Department of Psychiatry, The First Affiliated Hospital, Zhejiang University School of Medicine, China; and The Key Laboratory of Mental Disorder's Management of Zhejiang Province, China; Guangdong Mental Health Center, Guangdong Provincial People's Hospital, Guangdong Academy of Medical Sciences, Guangzhou, Guangdong province, China; and School of Medicine, South China University of Technology, Guangzhou, Guangdong province, China; Department of Psychiatry, Mental Health Center of Sichuan Province, China; Department of Psychiatry, Jining Mental Hospital, China; Department of Neurology, Xuanwu Hospital, Capital Medical University, China; Department of Radiology, China-Japan Friendship Hospital Affiliated to the Ministry of Health of PRC, China; Institute of Mental Health, The Sixth Hospital of Peking University, China; and Key Laboratory of Mental Health, Ministry of Health & National Clinical Research Center for Mental Disorders (Peking University), China; Institute of Mental Health, The Sixth Hospital of Peking University, China; and Key Laboratory of Mental Health, Ministry of Health & National Clinical Research Center for Mental Disorders (Peking University), China; Institute of Mental Health, The Sixth Hospital of Peking University, China; and Key Laboratory of Mental Health, Ministry of Health & National Clinical Research Center for Mental Disorders (Peking University), China; Institute of Mental Health, The Sixth Hospital of Peking University, China; and Key Laboratory of Mental Health, Ministry of Health & National Clinical Research Center for Mental Disorders (Peking University), China; Guangdong Mental Health Center, Guangdong General Hospital, China; and School of Medicine, South China University of Technology, Guangzhou, Guangdong province, China; Hebei Mental Health Center, The Sixth People's Hospital of Hebei Province, China; Henan Mental Hospital, The Second Affiliated Hospital of Xinxiang Medical University, China; Department of Neurology, Xuanwu Hospital, Capital Medical University, China; Shanghai Mental Health Center, Shanghai Jiaotong University, China; Mental Health Center, West China Hospital, Sichuan University, China; HuiLongGuan Clinical Medical School, Beijing HuiLongGuan Hospital, Peking University, China; Department of Psychiatry, First Hospital/First Clinical Medical College of Shanxi Medical University, China; Institute of Mental Health, The Sixth Hospital of Peking University, China; Key Laboratory of Mental Health, Ministry of Health & National Clinical Research Center for Mental Disorders (Peking University), China; and Peking-Tsinghua Joint Center for Life Sciences, IDG/McGovern Institute for Brain Research, Peking University, China; Institute of Mental Health, The Sixth Hospital of Peking University, China; and Key Laboratory of Mental Health, Ministry of Health & National Clinical Research Center for Mental Disorders (Peking University), China

**Keywords:** Antipsychotics, clinical trial, schizophrenia, drug interactions and side-effects, genetics

## Abstract

**Background:**

Schizophrenia is a severe and complex psychiatric disorder that needs treatment based on extensive experience. Antipsychotic drugs have already become the cornerstone of the treatment for schizophrenia; however, the therapeutic effect is of significant variability among patients, and only around a third of patients with schizophrenia show good efficacy. Meanwhile, drug-induced metabolic syndrome and other side-effects significantly affect treatment adherence and prognosis. Therefore, strategies for drug selection are desperately needed. In this study, we will perform pharmacogenomics research and set up an individualised preferred treatment prediction model.

**Aims:**

We aim to create a standard clinical cohort, with multidimensional index assessment of antipsychotic treatment for patients with schizophrenia.

**Method:**

This trial is designed as a randomised clinical trial comparing treatment with different kinds of antipsychotics. A total sample of 2000 patients with schizophrenia will be recruited from in-patient units from five clinical research centres. Using a computer-generated program, the participants will be randomly assigned to four treatment groups: aripiprazole, olanzapine, quetiapine and risperidone. The primary outcomes will be measured as changes in the Positive and Negative Syndrome Scale of schizophrenia, which reflects the efficacy. Secondary outcomes include the measure of side-effects, such as metabolic syndromes. The efficacy evaluation and side-effects assessment will be performed at baseline, 2 weeks, 6 weeks and 3 months.

**Results:**

This trial will assess the efficacy and side effects of antipsychotics and create a standard clinical cohort with a multi-dimensional index assessment of antipsychotic treatment for schizophrenia patients.

**Conclusion:**

This study aims to set up an individualized preferred treatment prediction model through the genetic analysis of patients using different kinds of antipsychotics.

Schizophrenia is a severe psychiatric disorder with high heritability and multigenic inheritance, affecting approximately 1% of the world population.^1^ Antipsychotic drugs are the mainstay of acute and long-term schizophrenia treatment, but the treatment response and tolerability are highly variable.^2^ There are high rates of treatment discontinuation because of efficacy and tolerability issues.^3^ First-generation antipsychotics are often accompanied by significant side-effects, including extrapyramidal symptoms and tardive dyskinesia. Second-generation antipsychotics are associated with a variety of metabolic side-effects, such as dyslipidaemia, elevated glucose levels and weight gain, and may cause clinical exacerbation or psychotic relapse, often resulting in hospital stays and placing great burden on patients and their families. Antipsychotics represent a considerable fraction of healthcare costs in most developed countries.^4,5^ A modest improvement in outcomes may provide great benefits for society. Therefore, it is important to individualise drug treatment to obtain an adequate effect and minimise side-effects.

Several twin and family studies suggest that the response to antipsychotic treatment is a heritable trait. Studies on monozygotic twins observed a similar response to treatment with antipsychotics and similar levels of antipsychotic-induced weight gain.^6,7^ Given that schizophrenia has a high heritability, it is likely that there is a substantial genetic component to individual differences in treatment response.^8,9^

Pharmacogenetics research has focused on the identification of genetic variants contributing to individual variability regarding several antipsychotic-related phenotypes. In past decades, research has succeeded in identifying genetic variants associated with variability in antipsychotic treatment.^10–17^ These studies focused on encoding drug targets (pharmacodynamic candidates) or involvement in the metabolism of the drug itself (pharmacokinetic candidates). In contrast to candidate gene-based methods, genome-wide association studies (GWAS) could identify candidate variants without introducing prior hypothesis bias. Several groups have performed GWAS to identify candidate biomarkers for treatment response and side-effects of antipsychotics. Currently, research has mainly used the genotyping data from the original Clinical Antipsychotic Trials of Intervention Effectiveness (CATIE) study, and focused on various phenotypes.

However, the vast majority of common variants associated with treatment response and side-effects of antipsychotics were identified only in the samples of European ancestry.^18–22^ The associated variants identified in populations of European ancestry might not be significant in other ancestry groups, because of underlying genetic heterogeneity. Therefore, there is a need for larger studies on the Chinese Han population, and independent samples to verify if there is any common genetic mechanism at the treatment response of the cross-ethnic population.

## Study objectives

There are three primary objectives of this study. First, we aim to create a standard clinical cohort with multidimensional index assessment of antipsychotic treatment for patients with schizophrenia, which can be dynamically monitored. Second, we aim to perform the genetic association analysis with the standard clinical cohort, to identify the risk factors and biomarkers of drug efficacy and side-effects. Finally, we will conduct further research on different antipsychotics, to construct a multidimensional evaluation index system for treatment with antipsychotics and set up an individualised prediction model to help drug selection and improve treatment efficacy, thereby reducing side-effects and improving long-term prognosis.

## Method

### Design and setting

The study on individualised optimal treatment for antipsychotic drugs is a parallel, double-blind, randomised, controlled multicentre study, conducted from July 2016 to December 2020. Patients will be enrolled from five research centres (the Sixth Hospital of Peking University, the First Hospital of Shanxi Medical University, Shanghai Mental Health Centre, West China Hospital of Sichuan University and Beijing HuiLongGuan Hospital), containing 15 hospitals (Capital Peking University Sixth Hospital, the First Hospital of Shanxi Medical University, Shanghai Mental Health Centre, West China Hospital of Sichuan University, Beijing HuiLongGuan Hospital, Medical University Xuanwu Hospital, Henan Psychiatric Hospital, Nanjing Brain Hospital, The First Affiliated Hospital of Medical School of Zhejiang University, Guangdong Mental Health Center, Hebei Mental Health Center, Wuxi Mental Health Center, Sichuan Mental Health Center, Jining Psychiatric Hospital, China-Japan Friendship Hospital), across China. Considering the importance of coherence, we have conducted five pieces of training for 104 psychiatrists in 15 hospitals, respectively. The training included research protocols, standard diagnostic criteria and other scales for the assessment of symptoms and side-effects, as well as videos for standardised scale assessments and blood sample collections.

We will perform several assessments of baseline status at the start of the study. Then, patients who meet the criterion will be randomly assigned (1:1:1:1) to four groups (aripiprazole, olanzapine, quetiapine and risperidone). Group assignment will be determined by a Microsoft Excel (Excel 2016 for Windows) randomisation generator, without any stratification factors. The random allocation sequence will be generated by a trained research assistant, and will be concealed until after the baseline assessments. In detail, 200 cases were taken as a block group, and the research participants were randomly grouped according to the block randomisation method. We generated the random numbers with the Microsoft Excel formula (ROUNDUP (RAND ()*10 000,0)) as integers between 0 and 9999. A patient ID is a five-digit number generated from the centre number and random number.

**Fig. 1:**
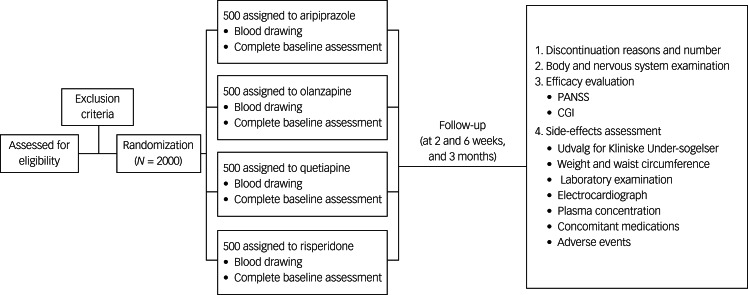
Flowchart of this study. CGI, Clinical Global Impressions Scale; PANSS, Positive and Negative Syndrome Scale.

All of the researchers administering baseline and follow-up assessments will be masked to the group assignments of each participant. All of the patients will be followed for up to 3 months or until the treatment was discontinued for any reason. Figure 1 shows a schematic diagram of the study design.

The trial is designed to use a standard clinical cohort to find out the risk factors and biomarkers of efficacy and side-effects of different drugs, which can help us establish individualised preferred treatment prediction model.

### Participants

We will enrol a sample of 2000 Chinese Han descents aged 18–45 years, who currently meet or have met the DSM-IV diagnostic criteria for schizophrenia or schizophreniform disorder based on the Structured Clinical Interview for DSM-IV, a review of their clinical records and input from available informants. The consensus diagnoses will be made by at least two experienced psychiatrists. Inclusion and exclusion criteria are listed in Tables 1 and 2. Informed consent will be obtained at the outset of the study. All participants will be asked to appoint a family member or close friend who is involved with the informed consent discussion and assists the patient with decision-making. The power analysis was performed by G*Power (version 3.1 for Windows, Heinrich Heine University Düsseldorf, Germany; see http://www.gpower.hhu.de/), and this sample size has achieved a power of 0.9.

**Table 1:** Inclusion criteria

Patients must be 18–45 years of age and of Han Chinese descent.
Patients must receive a diagnosis of schizophrenia, as determined based on the Structured Clinical Interview of the DSM-IV.
Total score on the Positive and Negative Syndrome Scale (PANSS) of >60, and at least three positive items were scored >4.
Patients must be physically healthy and have all laboratory parameters within normal limits.
Patients entering the study must have a condition appropriate for treatment with an oral medication.
Patients must provide informed consent to participate. All participants are asked to appoint a family member or close friend who is involved with the informed consent discussion and assists the patient with decision-making.

**Table 2:** Exclusion criteria

Patients with a DSM-IV diagnosis of schizoaffective disorder, mental retardation, pervasive developmental disorder, delirium, dementia, amnesia or other cognitive disorders are excluded.
Patients with severe unstable physical diseases, such as diabetes, thyroid diseases, hypertension and cardiac diseases, are excluded.
Patients with a diagnosis of narrow angle glaucoma are excluded.
Patients with a well-documented history of epilepsy or hyperpyretic convulsion are excluded.
Patients with a DSM-IV diagnosis of alcohol or drug dependence are excluded.
Patients who require long-acting injectable medication to maintain treatment adherence are excluded.
Patients regularly treated with clozapine over the past month are excluded. Patients who have taken clozapine for reasons other than treatment resistance may be eligible.
Patients who treated with electroconvulsive therapy over the past month are excluded.
Patients with a history of drug-induced malignant syndrome are excluded.
Patients with a history of serious suicide attempt, or excited agitation are excluded.
Patients with the following abnormities upon liver or renal function examination are excluded: aspartate transaminase ≥80 U/L, alanine transaminase ≥80 U/L, blood urea nitrogen ≥9.75 mmol/L, urine creatinine ≥21.6 mmol/d.
Patients who have no legal guardian are excluded.
Patients with corrected QT interval (QTc) prolongation (screening electrocardiogram with QTc > 450 msec for men, QTc > 470 msec for women) are excluded.
Women who are pregnant or breastfeeding are excluded.
Patients with well-documented histories of failure to respond to even one of the proposed treatment arms are excluded. A treatment failure has occurred if the patient continues to demonstrate severe psychopathology despite fully adhering to treatment at an adequate dose of the medication for an appropriate length of time.
Patients with a contraindication to any of the drugs to which they might be assigned are excluded.

### Pharmacological treatments

All patients will receive 6-week monotherapy of four kinds of randomly assigned antipsychotic drugs (aripiprazole, olanzapine, quetiapine and risperidone). Clinicians can prescribe the drugs based on individual patients, therapeutic response and side-effect burden (risperidone 2–6 mg/d, olanzapine 5–20 mg/d, quetiapine 400–750 mg/d and aripiprazole 10–30 mg/d). Withdrawal of the original medication and dosage titration to the lowest therapeutic dose will occur within 1 week. According to patients’ adverse reactions and drug efficacy, clinicians may continue to adjust the dose within the first 2 weeks, but the scope should be within the prescribed dosage. After the first 2 weeks, the dosage should not change during the study period.

### Concomitant medications

All participants will be maintained on the same antipsychotic medication throughout the entire study period, as medically feasible, with no introduction of new chronic therapies. Data will not be analysed from participants who make changes to the type of treatment they are receiving during the 6-week study period. However, data from participants who change the dose of their medication, without changing the type, will be analysed.

Other psychotropic medications, including antidepressants, anxiolytics and mood stabilisers, are not allowed during the study period. Patients who receive electroconvulsive therapy should be excluded. For 2 weeks before enrolment and throughout the study period, patients should not take drugs that will induce or inhibit drug-metabolising enzymes, such as rifampin, carbamazepine, phenobarbital and phenytoin. During the study, clinicians should minimise the use of aspirin and non-steroidal anti-inflammatory drugs. Patients who receive systemic psychotherapy will also be excluded.

In the following cases, after clinicians have confirmed the specific conditions, patients may receive combination medications. Patients with serious somnipathy may receive an intermittent short-term oral sedative-hypnotics of non-benzodiazepine and benzodiazepine medications in the evening (to include clonazepam, estazolam, lorazepam and others), but the duration of treatment should not exceed 14 days. When patients present with extrapyramidal symptoms, they may receive trihexyphenidyl according to the dosage instructions. If patients present with acute dystonia, they may be treated with scopolamine 0.3 mg each time, administered by intramuscular injection. If patients present with akathisia and tachycardia, they would be treated with beta-adrenoceptor blocking agents (such as propranolol and metoprolol). Treatment for somatic diseases may continue to be taken during the study period, but the dosage should preferably remain constant. General physical disease that occurs during the study may receive symptomatic treatment. When patients present with excitement or agitation symptoms, they may receive clonazepam (1–3 mg) by intramuscular injection. The daily dose should not exceed 6 mg, and the duration should not exceed more than a week. They may also be treated with oral lorazepam (1–2 mg) or clonazepam (1–2 mg); the daily dose should not exceed 6 mg and the duration should not exceed more than a week. To avoid affecting the outcomes, within 2 hours before each assessment, clinicians should prohibit the use of sedative-hypnotics or anti-Parkinsonian drugs.

### Discontinuation of participants

Participants might be discontinued from the study during the study period. We will record the reasons for their discontinuation as follows: (a) patients who violate the rules of the research project (e.g. patients who use banned drugs during the study, or patients who do not complete the study according to the protocol); (b) patients who have severe adverse effects or drug reactions; (c) patients who show poor adherence; (d) patients who withdraw their informed consent; (e) women who become pregnant during the study; (f) patients who fail to attend follow-up appointments; (g) according to the condition of patient, a psychiatrist will judge whether the patient should continue to participate in the study and can suspend those who are no longer suitable for the study because of reasons such as a lack of efficacy or intolerability; and (h) other reasons.

### Measurements

The participants should follow the schedule to attend the study visits for clinical examinations and to participate in the assessments (Table 3). Outcome measures are obtained from a variety of sources, including patient self-report, clinician ratings and ratings by trained study personnel.

**Table 3:** Study visit schedule

Assessment content	Screening and baseline (first interview)	14th day (second interview)	42nd day (third interview)	End of third month or early termination (fourth interview)
Informed consent	*			
Patient screening	*			
General information	*			
Symptoms and medical history	*			
Structured Clinical Interview for the DSM-IV-TR	*			
Body and nervous system examination	*	*	*	*
Vital signs, weight and waist circumference	*	*	*	*
PANSS and CGI-S^25^	*	*	*	*
UKU	*	*	*	*
Laboratory examination and ECG	*		*	*
Blood drawing for genotyping	*			
Plasma concentration			*	*
Concomitant medication monitoring	*	*	*	*
Adverse events	*	*	*	*
Form for the study end				

PANSS, Positive and Negative Syndrome Scale; CGI-S, Clinical Global Impressions Scale–Severity of Illness; UKU, Udvalg for Kliniske Under-sogelser Side-Effect Rating Scale; ECG, electrocardiogram.

#### Baseline measures

At baseline, participants should complete a form assessing demographic factors (e.g. gender, age, ethnicity, income, inhabiting information and education), symptoms and medical history. Participants will then go through the Structured Clinical Interview for DSM-IV-TR.^23^ The clinicians will also perform body and nervous system examination and monitor vital signs for patients. The Positive and Negative Syndrome Scale (PANSS)^24^ and the Clinical Global Impressions Scale–Severity of Illness (CGI-S)^25^ will be used to assess symptom severity in all participants. Then, a series of examinations will be performed, including laboratory examination, electrocardiogram, plasma concentration, concomitant medications monitoring and adverse events records. Finally, a blood sample will be obtained and stored for genotyping.

#### Efficacy evaluation

The efficacy of the antipsychotics will be evaluated with the PANSS (major index) or CGI-S (minor index) scales. The 30 items of the PANSS measure a broad range of symptoms typical for schizophrenia.^24^ The PANSS scores are labelled as positive, negative and general psychopathology subscale scores, along with the total symptom scores, which is the sum of the 30 items. The CGI-S rates the severity of schizophrenia symptoms on a scale from one to seven, with higher scores indicating greater severity of illness.^26^ All of the participants will receive these assessments at baseline, weeks 2 and 6, and the end of the third month (Table 3).

#### Side-effects and other assessments

Side-effects are assessed with the Udvalg for Kliniske Under-sogelser Side-Effect Rating Scale.^27–29^ In addition, to evaluate the effect of antipsychotic treatments on weight gain, glucose and lipid metabolism, each participant's pulse, blood pressure, fasting blood glucose level, haemoglobin, lipid profile (total cholesterol, high-density lipoprotein cholesterol and triglycerides) and weight will be assessed at baseline, weeks 2 and 6, and the end of the third month. Beyond that, complete blood count and serum prolactin level will be collected according to the schedule of events (Table 3).

### Genotyping and sequencing

Genomic DNA samples will be extracted with the QIAamp DNA Mini Kit (QIAGEN). Genotyping of samples will be conducted using the Illumina Global Screening Array (Illumina, San Diego, CA), which was designed to include a core imputation backbone (approximately 660 000 markers) and thousands of other custom add-on markers of interest concerning Alzheimer's disease, drug metabolism, fertility, twinning and schizophrenia (approximately 40 000 markers). Normalised bead intensity data for each sample will be loaded into Illumina BeadStudio software (Illumina, Sand Diego, US; see https://www.illumina.com.cn/), which can convert fluorescence intensities into single-nucleotide polymorphism (SNP) genotypes. The quality control will be performed before conducting the association analysis. Samples will be excluded according to the following criteria: genotype call rate of <98%, gender discordance, samples with an inbreeding coefficient (F_HET) of <−0.2 or >0.2, and genetic outliers. SNPs will be excluded according to the following criteria: minor allele frequency of <0.01, genotype call rate of <95% (before sample removal), SNP missingness of <98% (after sample removal) and *P*-values in Hardy–Weinberg equilibrium of <1 × 10^−6^ in cases. Principal component analysis will be performed to identify genetic outliers, following the methodology of the EIGENSTRAT (Harvard University and The Broad Institute, US; see https://github.com/chrchang/eigensoft/tree/master/EIGENSTRAT) software.^30^

Genotype imputation will be carried out for the discovery stage, using the pre-phasing/imputation stepwise approach.^31^ Genotypes will be first phased with SHAPEIT (Linux (x86_64), version 2 http://mathgen.stats.ox.ac.uk/genetics_software/shapeit/shapeit.html),^32^ and imputation then performed over each 3-Mb interval centred on all index SNPs, using IMPUTE2 (Linux (x86_64) Static Executable, https://mathgen.stats.ox.ac.uk/impute/impute_v2.html) software.^31^ Haplotypes derived from phase 1 of the 1000 Genomes Project (release v3, https://catalog.coriell.org/1/NHGRI/Collections/1000-Genomes-Collections/1000-Genomes-Project) will be used as reference data. We will use the same quality control, imputation and analysis pipeline for each GWAS in the discovery stage. We will exclude imputed SNPs with an imputation quality score below a set threshold (info <0.8), minor allele frequency of <0.01 and Hardy-Weinberg equilibrium *P*-value of  < 1 × 10^−5^. All genomic locations are given in National Center for Biotechnology (NCBI, https://www.ncbi.nlm.nih.gov/) Build 37 coordinates.

### Analysis plan

#### Primary outcomes

##### PANSS reductive ratio

We will use short-term (at 6-week follow-up) changes in the PANSS reductive ratio to indicate treatment effects of antipsychotics. To avoid incorrect calculations, the theoretical minimum (30 for the total score) is subtracted from the baseline score, resulting in a score range that includes zero. Therefore, PANSS percentage change was defined as follows: the reduction rate of the total score of PANSS = (PANSS baseline score − PANSS end-point score)/(PANSS baseline score − 30) × 100%.

#### Secondary outcomes

##### Other effects of antipsychotics

We will assess short-term (at 6-week follow-up) changes in side-effects in both groups, using measures such as body mass index, blood pressure, fasting glucose, haemoglobin and waist and hip circumference. We will also assess short-term (at 6-week follow-up) changes in the Udvalg for Kliniske Under-sogelser Side-Effect Rating Scale.

### Statistical analysis

Repeated measurements over time will be compared among the four groups, with the linear mixed model. The first analysis is an investigation into the relationship between the common variants and the efficacy of acute-stage treatment with different antipsychotics. We use the PANSS reductive ratio as the evaluation of acute-stage treatment response to different antipsychotic medications. We also use change in CGI-I score to assess the acute-stage treatment efficacy. Using PLINK version 1.07 (for Linux, https://www.cog-genomics.org/plink2),^33^ we will evaluate the association between allele dosages and the quantitative phenotype by the linear regression model. Gender, age, site of collection and the first four principal components of population structure will be used as covariates.

The second analysis of the secondary outcome consists of an investigation into the associations between the common variants and the side-effects of different antipsychotics. We will evaluate two kinds of phenotypes for side-effects of antipsychotics: dichotomous phenotype and continuous phenotype. The dichotomous phenotype will comprise patients with metabolic syndromes versus patients without metabolic syndromes. Metabolic syndrome diagnoses are based on the International Diabetes Federation Chinese criteria.^34^ Patients are identified as having a metabolic syndrome if they have central obesity (as assessed by waist circumference ≥85 cm in men and ≥80 cm in women), plus two of the following: elevated triglycerides level of ≥150 mg/dL (or use of a fibrate), high-density lipoprotein <40 mg/dL in men and <50 mg/dL in women (or use of a statin), fasting glucose ≥100 mg/dL (or use of an antidiabetic drug) and systolic arterial blood pressure ≥130 mmHg and/or diastolic arterial blood pressure ≥85 mmHg (or use of an antihypertensive drug). The continuous phenotype will comprise quantifying antipsychotic-induced change in the assessments as described above; for example, change in body mass index, blood lipids, glucose and haemoglobin. Using PLINK version 1.07,^33^ we will evaluate the association between allele dosages and the dichotomous phenotype by logistic regression, and perform the association between allele dosages and the quantitative phenotype by linear regression. The gender, age, site of collection and the first four principal components of population structure will be used as covariates.

The individualised preferred treatment prediction model will be set up based on the findings of different antipsychotics and the clinical data.

#### 

##### Ethics and dissemination

The study was reviewed and approved by the institutional ethical committee of each hospital. The study has been registered with Chinese Clinical Trial Registry, under registration number ChiCTR1800014755.

## Discussion

There are several strengths to our study. The main strength is that it can provide a criterion to create a standard cohort, so that data can be well-preserved and used in later studies. A further strength is that this study is, to our knowledge, the first of its kind to be conducted on such a large scale among the Chinese Han population. Also, it considers both the efficacy and side-effects of antipsychotics. A limitation of this study is that it had a follow-up period of only 3 months.

In conclusion, this protocol describes the aims, hypotheses, design and procedures of the clinical trial, and its proposed method of identifying genetic determinants of response and side-effect to acute-stage antipsychotics treatment in patients with schizophrenia. This study may provide important evidence for translating the valuable findings from pharmacogenomics studies into clinical practice.

## Data Availability

The data that support the findings of this study are available from the corresponding author, W.Y., upon reasonable request.
